# Targeting the programmed cell death signaling mechanism with natural products for the treatment of acute pancreatitis: a review

**DOI:** 10.3389/fphar.2025.1567552

**Published:** 2025-05-30

**Authors:** Xuehuan Liu, Baolei Dou, Qingjun Zhu, Chuanguo Liu

**Affiliations:** ^1^ The Experimental Center, Shandong University of Traditional Chinese Medicine, Jinan, China; ^2^ College of Traditional Chinese Medicine, Shandong University of Traditional Chinese Medicine, Jinan, China; ^3^ Innovative Institute of Chinese Medicine and Pharmacy, Shandong University of Traditional Chinese Medicine, Jinan, China; ^4^ Key Laboratory of Traditional Chinese Medicine Classical Theory, Ministry of Education, Jinan, China

**Keywords:** acute pancreatitis, natural products, programmed cell death, signaling pathways, therapy

## Abstract

Acute pancreatitis (AP) is a common critical disease in clinical practice, characterized by acute onset, rapid progression, aggressive conditions, and high lethality. Pancreatic acinar cell death is a central event in the pathological process of AP and a key factor in determining the extent of local or systemic inflammatory injury and overall prognosis. Programmed cell death (PCD) is a form of active cell death regulated by multiple genes, including apoptosis, autophagy, ferroptosis, pyroptosis, and necroptosis. PCD plays a critical role in eliminating unwanted organisms and damaged cells, which is of great significance. Numerous studies have demonstrated a strong association between various forms of PCD and AP, and targeted interventions in PCD signaling pathways and key targets can influence the progression of AP. Furthermore, existing research indicates that natural products sourced from plants, fruits, and vegetables exhibit considerable potential in targeting and regulating PCD for the treatment of AP. Therefore, this paper focuses on summarizing the common types of PCD in AP and discusses the specific signaling pathways and key targets reported in the treatment of AP using natural products. This review aims to provide a reference for natural products in guiding AP treatment and to lay the foundation for developing new drugs to effectively prevent and manage AP.

## 1 Introduction

Acute pancreatitis (AP) is an inflammatory condition caused by the activation of pancreatic enzymes, resulting in pancreatic autodigestion. Its hallmark symptom is the acute onset of severe, persistent epigastric pain, frequently accompanied by abdominal distension, nausea, and vomiting (Xiao et al., 2022). AP generally exhibits a self-limiting course, with the majority of cases presenting as mild. Nevertheless, approximately 20% of patients progress to severe acute pancreatitis (SAP), which is frequently associated with multiple organ dysfunction syndrome and systemic inflammatory response syndrome, thereby substantially elevating the risk of mortality (Leppäniemi et al., 2020). Currently, AP treatment relies on fluid resuscitation, symptomatic management, and supportive care, as no targeted pharmacological therapies are available. The efficacy of pancreatic enzyme inhibitors and growth inhibitors remains unverified, and antibiotic use is highly controversial (Szatmary et al., 2022). Thus, identifying new therapeutic strategies is an urgent priority. Programmed cell death (PCD) is a genetically regulated, active, and orderly process of cell death, including apoptosis, autophagy, ferroptosis, pyroptosis, and necroptosis. PCD is fundamental to organismal evolution, maintaining homeostasis, and the development of various tissues and organs (Chen Y. et al., 2024). Research has indicated that PCD is integral to the pathogenesis and advancement of AP and that targeted modulation of PCD-related signaling pathways can mitigate pancreatic tissue damage and ameliorate AP symptoms. In recent years, natural metabolites have exhibited efficacy and safety in preventing and treating tumors, renal disorders, and cardiovascular diseases, owing to their multi-component and multi-target characteristics (Avila-Carrasco et al., 2021; Cai J. et al., 2021; Vahdat-Lasemi et al., 2021). Certain natural products have also shown considerable promise in preventing and treating AP. These metabolites can reduce the progression of AP and improve its prognosis by modulating various forms of PCD. Therefore, these natural products represent promising candidates for the therapeutic management of AP. This article offers a comprehensive overview of the diverse forms of PCD related to AP, examines the principal targets that regulate PCD within the context of AP, and underscores the efficacy of utilizing natural products (e.g., bioactive phytochemicals and dietary metabolites) to target PCD for the treatment of AP.

## 2 Methods

### 2.1 Search strategy

This study collected relevant literature on natural products targeting PCD for AP treatment through systematic searches of scientific databases. The search spanned from January 2015 to January 2025, restricted to English-language articles, and covered PubMed and Web of Science. Duplicates were removed using EndNote X20, and literature was managed with Mendeley. The search strategy integrated AP-related terms (“acute pancreatitis” OR “AP”), PCD subtypes (“programmed cell death,” “apoptosis,” “necroptosis,” “pyroptosis,” “ferroptosis”), natural product descriptors (“natural products,” “phytochemicals,” “herbal extracts,” “plant-derived”), and mechanistic terms (“signaling pathways,” “therapeutic targets,” “mechanisms”). Additional terms such as “pancreatic acinar cell death” were included to enhance search comprehensiveness.

### 2.2 Inclusion and exclusion criteria

Studies were screened according to predefined inclusion and exclusion criteria. Inclusion criteria required articles to: 1) focus on natural products targeting PCD in AP treatment, with original research involving cell/animal experiments or clinical trials; 2) explicitly analyze PCD mechanisms; and 3) report therapeutic outcomes such as reduced pancreatic necrosis, decreased amylase/lipase levels, or suppressed inflammatory cytokines. Exclusion criteria comprised: 1) non-experimental articles (e.g., reviews, network pharmacology studies, conference abstracts); 2) non-bioactive phytochemicals and dietary metabolites (e.g., animal/microbial extracts); 3) studies not targeting PCD in pancreatic acinar cells; and 4) lack of PCD mechanistic analysis. To ensure reliability, two reviewers independently screened titles/abstracts and assessed full texts, with discrepancies resolved by a third reviewer.

### 2.3 Data extraction and analysis

**FIGURE 1:**
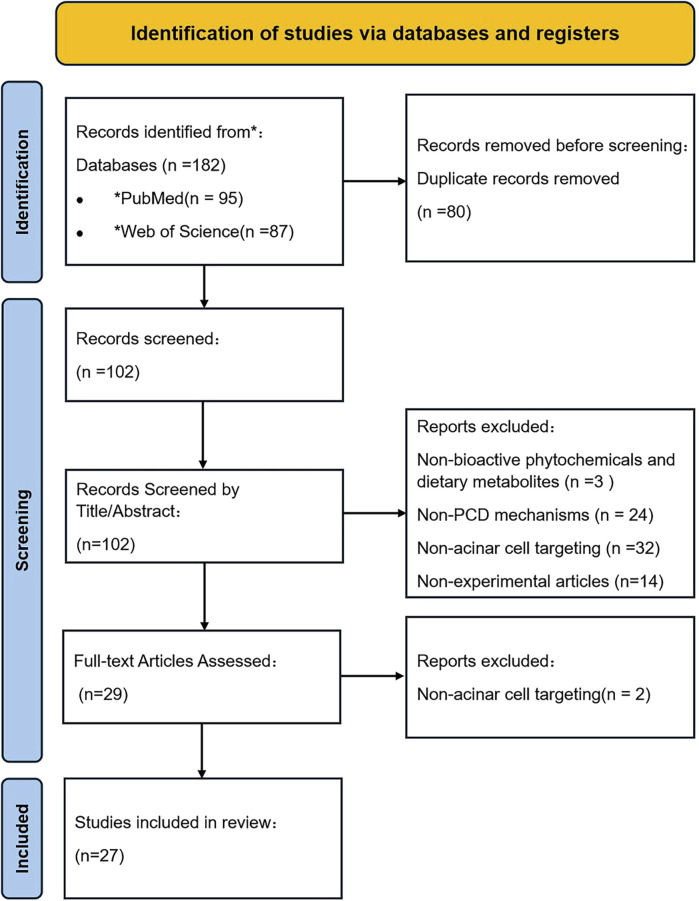
PRISMA flowchart depicting the literature management.

Data from eligible studies were systematically extracted, organized, and analyzed. Key parameters included natural product identity, structure, *in vitro*/*in vivo* models, doses, duration, controls, key altered factors, mechanisms, and references. The results of the data assessment are summarized in Tables 1 and 2, while the study selection process is illustrated in Figure 1.

**TABLE 1:** Natural products targeting apoptosis in AP.

Natural products	Structure	Type of study	Models	Doses	Duration	Controls	Altered factors	Mechanism	References
Nimbolide	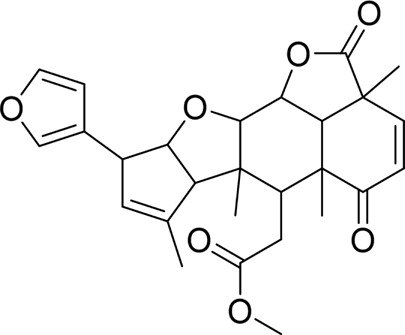	*In vivo*	AP model mice	0.3, 1 mg/kg	7 d	Saline	IL-1β↓IL-6↓TNF-α↓MDA↓GSH↑Bcl2↑Bax↓ cleaved - caspase-3↓	NF-κB↓	Bansod and Godugu. (2021)
Crocetin	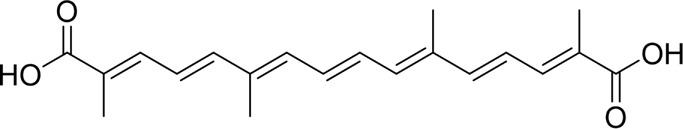	*In vitro*	AR42J cells	5, 10, 25, 50 μM	24 h	Not mentioned	IL-1β↓IL-6↓TNF-α↓Bcl-2↑Bax↓cleaved caspase −3↓CytoC↓	NF-κB↓	Zhu and He. (2022)
Total flavonoids of Chrysanthemum indicum L		*In vitro* and *in vivo*	AP model rats	300 mg/kg	3 d	Saline	IL-1β↓TNF-α↓IL-6↓IL-10↑p-p65↓Bax↓cleaved caspase-3↓Bcl-2↑	NF-κB↓	Yang et al. (2024)
			AR42J cells	50 mg/L	24 h	PBS			
Daphnetin	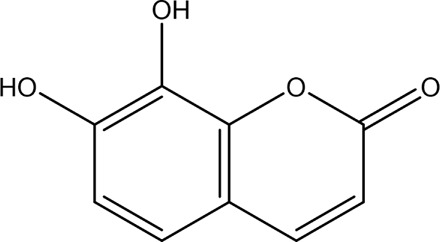	*In vivo*	SAP model rats	4 mg/kg	12 h	Not mentioned	SOD↑IL-10↑NF-κBp65↓apoptosis-rate↓	TLR4/NF-κB↓	Liu et al. (2016b)
Tetrandrine	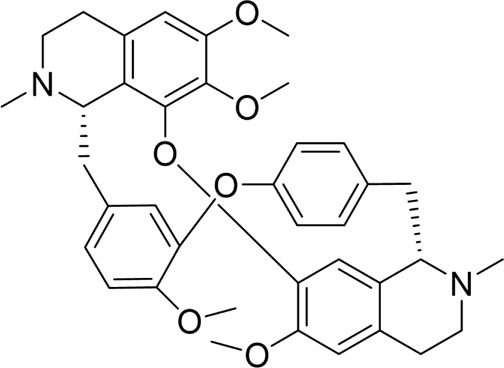	*In vivo*	SAP model rats	40 mg/kg	12 h	Saline, verapamil (1 mg/kg)	MPO↓NF-κB transporter activation rates ↓	NF-κB↓	Wu et al. (2015)
Anisodamine	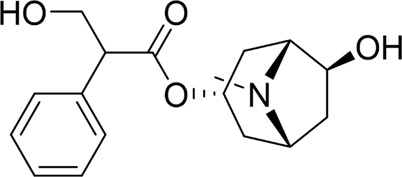	*In vitro*	AR42J cells	1, 10, 100, 200 μg/mL	48 h	Not mentioned	TNF-α↓IL-1β↓IL-18↓p-p65↓ p-IjBa↓	NF-κB↓	Li et al. (2020b)
Rutaecarpine	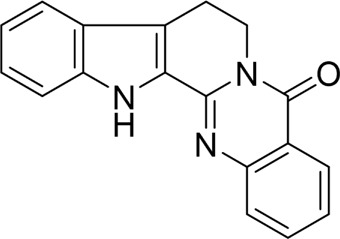	*In vitro* and *in vivo*	AP model mice	25, 50, 100 mg/kg	3 d	Saline	IL-6↓TNF-α↓IL-10↑p-JNK↓p-ERK↓p65↓p-IKBα↓p-IKKβ↓	MAPK and NF-κB↓	Huang et al. (2021)
			AR42J cells	70 μg/mL	6 h	Not mentioned			
Nobiletin	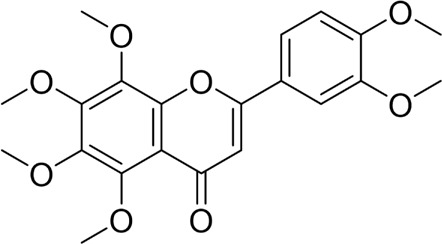	*In vivo*	AP model mice	25, 50 mg/kg	14 d	Saline	IL-1β↓IL-6↓TNF-α↓IL-10↑ROS↓cleaved caspase-3↓p-P38MAPK↓p-AKT↓	p38MAPK↓	Liu et al. (2016a)
Ligustrazine	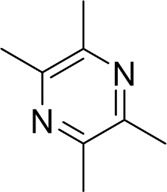	*In* *vitro* and *in vivo*	AP Model rats	150 mg/kg	72 h	Saline	TNF-α↓IL-1β↓IL-6↓MPO↓p53↑cleavedcaspase-3↑p-p38↓p-Erk↓	p38 and Erk MAPK↓	Chen et al. (2016)
			AR42J cells	1.0 mg/mL	12 h	Saline, p38 inhibitor SB203580(10 mM), Erk inhibitor PD98059 (100 mM)			
Escin Sodium	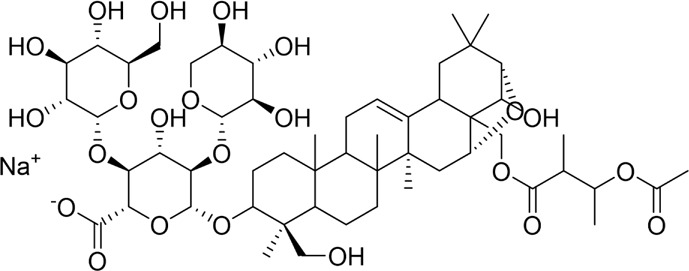	*In vitro* and *in vivo*	AP Model rats	1, 3, 6 mg/kg	25 h	Saline	TNF-α↓IL-6↓IL-1β↓IL-18↓IL-10↑MPO↓Bcl-2/Bax↓cleaved-caspase-3↑P53↑p-ERK↓p-STAT3↓	ERK/STAT3↓	Zhang et al. (2021a)
			AR42J cells	10, 20, 30 μg/mL	9 h	Erk inhibitor PD98059 (10 μM)			
Limonin	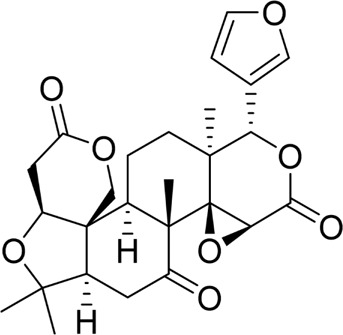	*In vitro* and *in vivo*	MAP model mice	25, 50, 100 mg/kg	12 h	Not mentioned	IL-6↓IL-1β↓CCL2↓TNF-α↓GSH↑SOD↑Cyclin D1↑Bcl-2↑	JAK2/STAT3↓	Xia et al. (2021)
			SAP model mice	25, 50, 100 mg/kg	72 h				
Baicalin	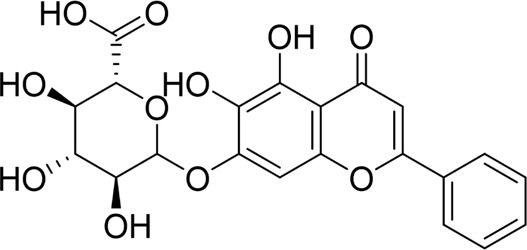	*In vivo*	SAP model mice	20 mg/kg	9 h	Saline	Bax↓cleaved caspase-3↓Bcl-2↑IL-6↓TNF-α↓IL-1β↓ROS↓NOX2↓SOD2↑	B7H4/JAK2/STAT3↓	Yang et al. (2023)
Genistein	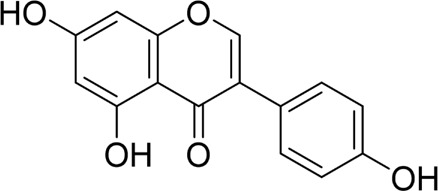	*In vivo*	AP model mice	5 mg/kg	10 h	Not mentioned	GRP78↑PERK↑eIF2a↑caspase-12↑CHOP↑	ERS↑	Xia et al. (2019)
Quercetin 3-O-xyloside	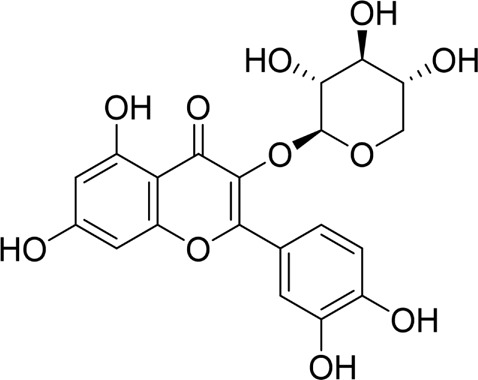	*In vitro*	AR42J cells	10, 50, 100 µM	8 h	Not mentioned	ROS↓GRP78↓PERK↓caspase-3↑caspase-9↑	ERS↓	Seo et al. (2019)
Lycopene	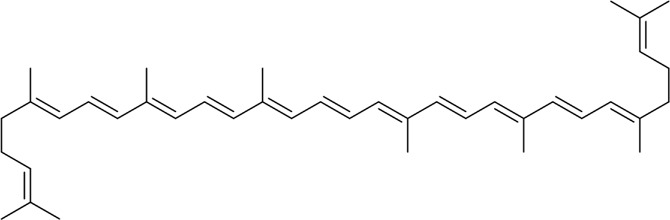	*In vitro* and *in vivo*	SAP Model rats	10 mg/kg	3 d	Sunflower oil (2 mL)	TNF-α↓IL-6↓MIP-1α↓MCP-1↓COX-2↓NF-κB p65↓SOD↑ROS↓cleaved caspase-3↓	ERS↓	Lv et al. (2015)
			AR42J cells	2.10 μM	24 h	PBS			
Piperine	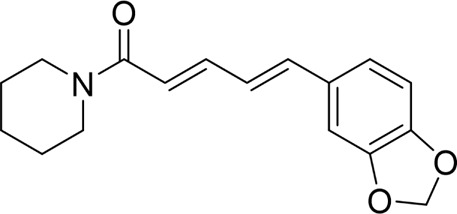	*In vivo*	AP model mice	25, 50, 75 mg/kg	48 h	4-phenylbutyrate sodium salt (40 mg/kg), rapamycin (10 mg/kg), 5-3-methyladenine (5 mg/kg), tunicamycin (2 mg/kg)	p-PERK↓ p-eIF2α ↓Bax↓Cleaved caspase-3↓Bcl-2↑	ERS↓	Huang et al. (2022a)

↑: Activation/upregulation; ↓: Inhibition/downregulation.

**TABLE 2:** Natural products targeting autophagy, pyroptosis and ferroptosis in AP.

Programed cell death	Natural products	Structure	Type of study	Models	Doses	Duration	Controls	Altered factors	Mechanism	References
Autophagy	Salidroside	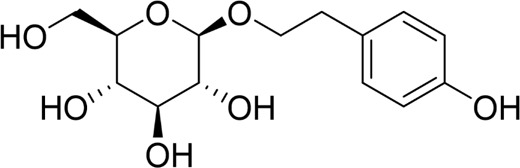	*In vitro*	AR42J cells	83, 167, 333, 666, 1332 μM	24 h	Not mentioned	TNF-α↓IL-1β↓IL-8↓IL-6↓IL-10↑Beclin-1↓LC3-II↓LAMP↑p-p65/p65↓p-IκBα/IκBα↑	NF-κB↓	Qian et al. (2021)
	Salidroside	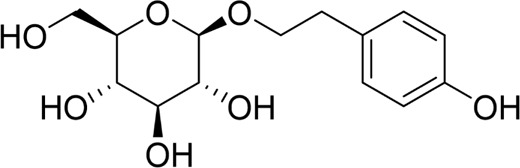	*In vivo*	SAP Model rats	5, 10, 20 mg/kg	24 h	Saline, pyrrolidine-dithiocarbamate (100 mg/kg)	TNF-α↓IL-1β↓IL-10↑ LC3II↓Beclin-1↓IκBα↑LAMP2↑	NF-κB↓	Qian et al. (2022)
	Acanthopanax	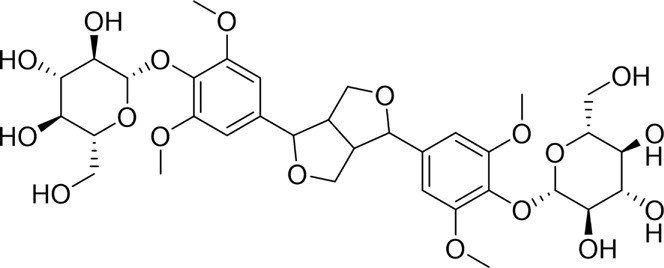	*In vivo*	SAP Model rats	3.5 mg/100 g	21 h	3- methyladenine (1.5 mg/100 g)	LC3-II↓Beclin-1↓ NF-κB p65↓	NF-κB↓	Wang et al. (2016)
	Phillygenin	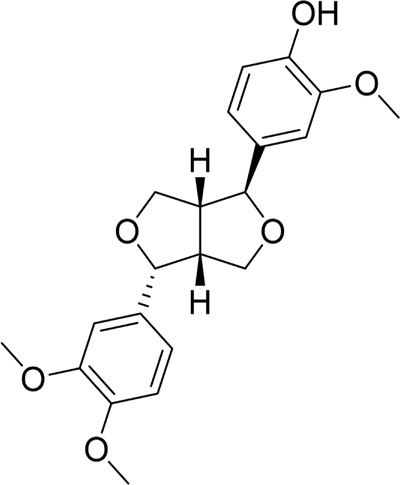	*In vivo*	SAP Model rats	30 mg/kg	12 h	Saline	LAMP-2↑p62↓p-PI3K↓p-Akt↓p-mToR↓	PI3K/Akt/mTOR↓	Li et al. (2024b)
	Xanthohumol	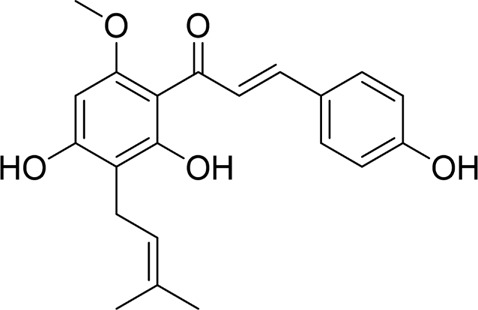	*In vitro* and *in vivo*	SAP model mice	3, 6, 9 mg/kg	25 h	Not mentioned	TNF-α↓IL-6↓IL-10↑ ROS↓MDA↓SOD↑Nrf2↑HO-1↑p62↓p-AKT↓LC3B-II/LC3B-I↓p-mTOR↓	AKT/mTOR↓	Huangfu et al. (2023)
				AR42J cells	15 μM	1 h				
	Mogroside IIE	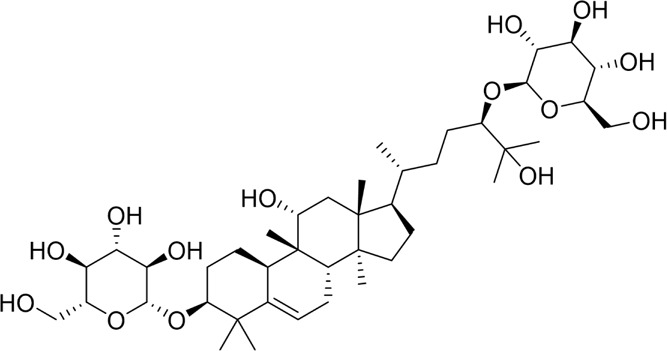	*In vitro* and *in* *vivo*	AP model mice	10 mg/kg	6 h	IL-9 (10 mg/kg)	p62↓LC3II↓IL-9↓	IL-9/IL-9receptor pathway↓	Xiao et al. (2020)
				AR42J cells	20 μM	13 h	Saline, 3-Methyladenine (2 mM), IL-9 (10 ng/mL)			
Pyroptosis	Sinapic acid	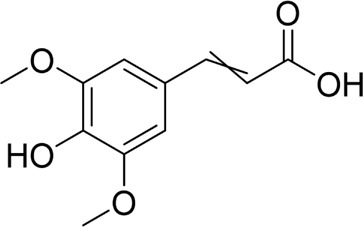	*In vivo*	AP model mice	10, 20, 40 mg/kg	40 h	Saline	TNF-α↓IL-6↓IL-1β↓Caspase-↓Caspase-11↓GSDMD↓NLRP3↓NF-κB↓IκB-α↑P-AMPK↓	AMPK/NF-κB↓	Huang et al. (2022c)
	Saikosaponin D	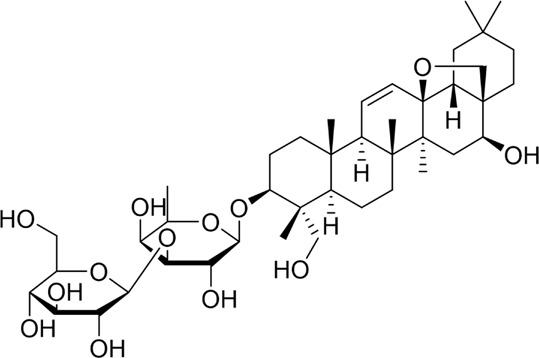	*In vitro*	AR42J cells	10, 20, 30 μM	24 h	Necrosulfonamide (10 μM), necrostatin-1 (10 μM), chloroquine (25 μM), z-Val-Ala-Asp(OMe)-fluoromethylketone (10 μM)	IL-1β↓IL-18↓MDA↓ROS↓SOD↑NLRP3↓Cleaved caspase-1↓	NLRP3/caspase-1↓	Chen et al. (2024a)
	Salidroside	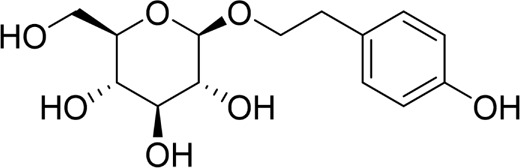	*In vitro* and *in* *vivo*	SAP Model rats	20 mg/kg	21 h	Not mentioned	IL-1β↓IL-18↓p-Akt↓cleaved caspase-3↓p-p65↓GSDME-N↓	Akt/NF-κB ↓ Caspase-3/GSDM↓	Wang et al. (2023c)
				AR42J cells	666 μM	24 h				
Ferroptosis	Wedelolactone	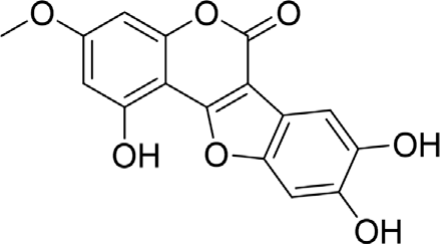	*In vitro* and *in vivo*	AP Model rats	25, 50 mg/kg	23 h	Saline, Ferrostatin-1 (2.5 μmol/kg)	GSH↑GPx↑GPX4↑MDA↓IL-1β↓IL-18↓	GPX4↑	Fan et al. (2021)
				AR42J cells	10, 20 μM	48 h	Deferoxamine (10 μM)			
	Lonicerin	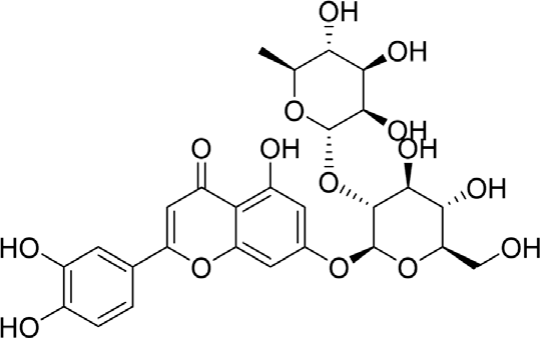	*In vitro*	AR42J cells	20, 40, 80 µM	48 h	Not mentioned	Fe^2+^↓MDA↓ROS↓GSH↑	SIRT1/GPX4↑	Li et al. (2024a)

↑: Activation/upregulation; ↓: Inhibition/downregulation.

## 3 PCD and AP

**FIGURE 2:**
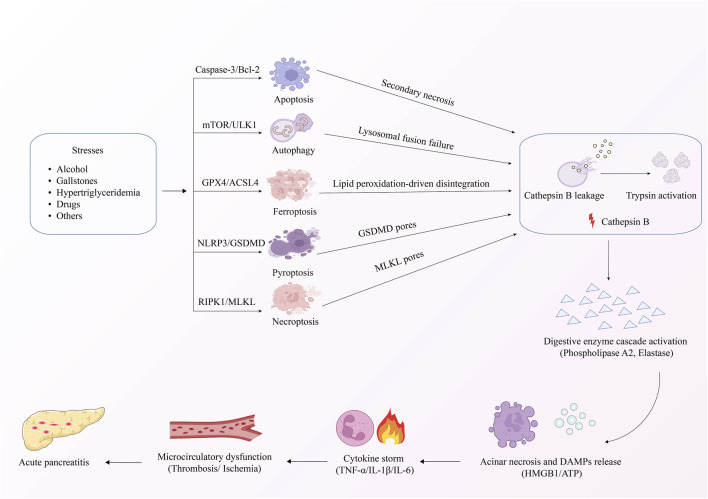
Mechanism of PCD in AP.

**FIGURE 3:**
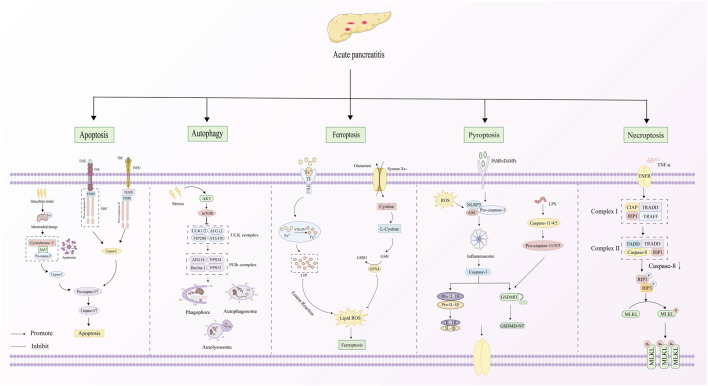
Signaling pathways o PCD in AP.

AP is distinguished by damage to pancreatic acinar cells, leading to the abnormal release and activation of trypsinogen within these cells. This process triggers a cascade involving the activation of digestive enzymes, along with the kinin and complement systems. Eventually, the pancreatic tissue is subjected to autodigestion (Mederos et al., 2021). Extensive research has established a critical link between AP pathogenesis mechanisms and PCD in pancreatic acinar cells (Figure 2). The PCD subtypes identified in pancreatic acinar cells include apoptosis, autophagy, ferroptosis, pyroptosis, and necroptosis (Li H. et al., 2023). The core signaling pathways governing these PCD mechanisms during AP progression are comprehensively summarized in Figure 3. The dual roles of all PCD types in AP are summarized in Table 3.

**TABLE 3:** Dual roles of PCD types in AP.

PCD type	Protective role	Pathogenic role	Regulation strategies	References
Apoptosis	Early clearance of damaged cells reduces inflammation	Excessive apoptosis releases DAMPs	Moderate induction (early) vs. inhibition (late)	Najenson et al. (2018), Lv and Jin. (2019)
Autophagy	Maintains homeostasis by degrading misfolded proteins	Impaired autophagic flux or hyperactivation causes injury	Enhance flux (early) vs. suppress overload (late)	Voronina et al. (2022), Hu et al. (2020)
Pyroptosis	No direct evidence reported	Activation of the NLRP3 inflammasome results in the release of IL-1β and IL-18	Blockade of NLRP3 activation; Inhibition of GSDMD pore formation	Al Mamun et al. (2022), Gao et al. (2021)
Ferroptosis	No direct evidence reported	Lipid peroxidation-induced membrane damage; GPX4 inhibition exacerbates AP	Suppression of lipid peroxidation; Restoration of GPX4 activity	Li et al. (2022b), Wei et al. (2024)
Necroptosis	Potential protective effects under specific conditions: RIPK3/MLKL deficiency may reduce compensatory necrosis despite exacerbating apoptosis.	Dominant pathogenic role: RIPK1-RIPK3-MLKL pathway activation triggers acinar cell membrane disruption and DAMP-driven systemic inflammation	Inhibition of RIPK1 kinase activity; Blockade of MLKL phosphorylation	Yang et al. (2021), Boonchan et al. (2021)

### 3.1 Apoptosis and AP

Apoptosis is a form of PCD that is essential for the maintenance of internal homeostasis (Li and Du, 2024). Upon stimulation by various factors, apoptosis-related signaling pathways are activated, resulting in a series of morphological changes in the cell, such as cell shrinkage, reduced volume, increased cytoplasmic density, and the compression of organelles and chromatin. Subsequently, the cell membrane forms vesicles that enclose fragmented nuclear material, which ultimately form apoptotic vesicles. Concurrently, cysteine-aspartic proteases (caspases) are activated, initiating a protease cascade that amplifies the apoptotic pathway, eventually culminating in PCD (Monier and Suzanne, 2021). The principal regulatory mechanisms of apoptosis encompass the extrinsic (death receptor-mediated) and intrinsic [mitochondrial and endoplasmic reticulum stress (ERS)-mediated] pathways (Mustafa et al., 2024). The extrinsic apoptotic pathway is activated through extracellular apoptotic signals, during which death ligands like FasL, TNF-related apoptosis-inducing ligand (TRAIL), and tumor necrosis factor (TNF) attach to their specific receptors, such as FasR, DR4, and TNFR1. This binding enables the death domain (DD) within the receptors to recruit adaptor proteins, including FADD and caspase-8, forming the death-inducing signaling complex (DISC). Once DISC is assembled, it activates caspase-8, which subsequently cleaves caspase-3 directly. This cleavage triggers a cascade of caspase activation, culminating in apoptosis (Raducka-Jaszul et al., 2020). In the mitochondrial pathway, cytochrome c (Cytoc) is released into the cytoplasm due to alterations in the outer mitochondrial membrane induced by apoptosis-stimulating substances. This process results in the activation of apoptotic protease activating factor-1 (Apaf-1) and caspase-9, leading to the formation of a complex cascade involving Cytoc, Apaf-1, and caspase-9. This cascade ultimately activates caspase-3, thereby initiating the execution phase of apoptosis (Bedoui et al., 2020). In addition, ERS may be induced by a diverse array of stimuli. Under stressful conditions, the endoplasmic reticulum undergoes damage, leading to the buildup of misfolded proteins and triggering the unfolded protein response (UPR). If the UPR remains active for an extended period, it can ultimately result in apoptosis (Li and Sun, 2024).

More studies are revealing a strong connection between pancreatic acinar cell apoptosis and the development of AP. Siriviriyakul et al. employed TUNEL staining to reveal a significant increase in the rate of acinar cell apoptosis in L-arginine-induced AP mice compared to a control group of normal mice. This finding suggests that acinar cell apoptosis may play a contributory role in the progression of AP (Siriviriyakul et al., 2022). Sun et al. explored the function and mechanism of circular RNA utrophin (circ_UTRN) in AP and found that circ_UTRN modulates disease progression by inducing apoptosis in pancreatic acinar cells (Sun et al., 2022b). Najenson et al. administered atrial natriuretic peptide to an AP rat model and observed an alleviation of AP symptoms, which was attributed to the induction of pancreatic acinar cell apoptosis and a reduction in inflammatory mediator expression (Najenson et al., 2018). Another study revealed that inducing pancreatic acinar cell apoptosis during AP may attenuate the inflammatory cascade response, conferring a protective effect against the disease (Luo et al., 2020). Collectively, these findings suggest that promoting pancreatic acinar cell apoptosis may alleviate the severity of AP. However, not all studies support this perspective, and the role of apoptosis in acute pancreatitis remains a subject of debate. Notably, the impact of apoptosis in pancreatic acinar cells varies with context and intensity. These cells release damage-associated molecular patterns (DAMPs) like histones, DNA, and heat shock proteins. If apoptosis outpaces the phagocytosis of DAMPs, their accumulation can worsen pancreatic injury due to their proinflammatory nature. This balance between DAMP production and clearance explains the conflicting results in studies on AP progression (Gaman et al., 2018). Certain experts have discovered that the release of high mobility group box 1, histones, and DNA by apoptotic pancreatic acinar cells exacerbates pancreatic damage and inflammation (Lv and Jin, 2019). Furthermore, Xu et al. indicated that taurocholate sodium-induced inflammatory responses in pancreatic tissues of AP rats were enhanced, with upregulation of Bax and caspase-3 and downregulation of Bcl2. They also found that vitamin B6 protected pancreatic function in AP rats by inhibiting pancreatic acinar cell apoptosis and inflammatory responses, thus reversing these molecular changes (Xu et al., 2024). These experimental results confirm that apoptosis may exacerbate pancreatic injury and inflammation, whereas its inhibition may alleviate AP. In summary, apoptosis of pancreatic acinar cells might serve a dual role in the development of AP. Nevertheless, whether acinar cell apoptosis exerts a positive or negative effect during AP remains incompletely understood, warranting further investigation.

The progression of AP is regulated by a number of signaling molecules linked to apoptosis. Nuclear factor-κB (NF-κB) is an intracellular transcription factor that regulates the expression of genes associated with inflammation, immunological responses, cell proliferation, and apoptosis (Khan et al., 2024). Studies have demonstrated that the NF-κB signaling pathway is intimately linked to the onset and progression of AP. Blocking the NF-κB pathway has been shown to reduce the release of inflammatory mediators and suppress pancreatic acinar cell death in cerulein-induced AP mice, which improves damage to the pancreas (Lv et al., 2020). The mitogen-activated protein kinase (MAPK) signaling pathway comprises serine/threonine kinases that convey signals from outside the cell to the nucleus, regulating cell growth, differentiation, and apoptosis (Guo et al., 2020). Guo et al. indicated via experimental studies on rats with sodium taurocholate (STC)-induced AP that the inhibition of the MAPK signaling pathway mitigates apoptosis and inflammatory infiltration in pancreatic acinar cells, which in turn hinders disease progression (Guo et al., 2021). The Janus kinase 2 (JAK2)/signal transducer and activator of transcription 3 (STAT3) is a multifaceted signaling cascade that regulates cytokine and growth hormone receptor signaling, facilitating the initiation and development of AP (Liu D. et al., 2022). Activation of the JAK2/STAT3 signaling pathway has been reported to induce apoptosis and exacerbate pancreatic damage in acinar cells in AP disease models (Fei et al., 2019). Furthermore, the ERS pathway is implicated in AP. ERS causes the accumulation of misfolded proteins in the endoplasmic reticulum, which activates the UPR, ultimately inducing apoptosis in pancreatic acinar cells (Zhou et al., 2020). These results show that the progression of AP is strongly associated with pancreatic acinar cell apoptosis, which is modulated by diverse signaling pathways and biological targets.

### 3.2 Autophagy and AP

Autophagy involves a series of pathways through which cells degrade and recycle damaged organelles, lipids, and long-lived proteins via lysosomal degradation, thereby maintaining cellular homeostasis and meeting energy demands essential for biological growth (Wang et al., 2024a). Under physiological conditions, autophagy degrades misfolded proteins and damaged organelles generated by cellular metabolism, facilitating cellular renewal and metabolic regulation. In pathological contexts such as nutrient deprivation, hypoxia, or oxidative stress, autophagy is upregulated to preserve cellular homeostasis (Ortega et al., 2024). Autophagy can be separated into three types according to substrate specificity and transport mechanisms: macroautophagy, microautophagy, and chaperone-mediated autophagy. These autophagy types transport damaged organelles and misfolded proteins to lysosomes through distinct mechanisms. Lysosomal degradation breaks down these substrates into amino acids and other by-products, which are then recycled into the cytoplasm for the synthesis of new macromolecules (Liu S. et al., 2023). Macroautophagy, hereafter known as autophagy, is the most extensively studied form among these types and is the only type observed in both the normal exocrine pancreas and pancreatitis (Gukovskaya et al., 2017). The autophagy process consists of three key stages: initiation, autophagosome membrane elongation, and autolysosome formation. This process requires the involvement of multiple autophagy-related genes (Atg) (Hu and Reggiori, 2022). The Atg1/Unc-51-like autophagy activating kinase 1 complex is critical for autophagy initiation and autophagosome formation (Backe et al., 2023). The class III phosphatidylinositide 3-kinase (PI3K) complex recruits autophagy-related proteins and is involved in the formation, maturation, and trafficking of autophagic vesicles (Safaroghli-Azar et al., 2023). Two ubiquitin-like systems, the Atg12-Atg5-Atg16 complex and Atg8/light chain 3 (LC3) family proteins, facilitate the elongation and formation of autophagosome membranes (Chen et al., 2019). Autophagosomes form and fuse with lysosomes through the coordinated action of Atg proteins, and their contents are subsequently degraded by lysosomal proteases (Matoba and Noda, 2021).

Research has proven the vital significance of autophagy in the pathophysiology of AP, though its role is profoundly context-dependent, dynamically shifting between protective and pathogenic effects based on spatiotemporal regulation, disease stage, and stressor intensity/duration. In early-stage AP, autophagy temporarily provides protective effects by removing damaged organelles, such as swollen mitochondria, and sequestering prematurely activated zymogens, such as trypsinogen, within autophagic vacuoles. This process represents a critical pathological feature associated with disease prognosis (Voronina et al., 2022), and it serves to delay the autodigestion of acinar cells. Under physiological conditions, moderate autophagy maintains cellular equilibrium by removing misfolded proteins and dysfunctional organelles (Antonucci et al., 2015; Ding et al., 2024). As acute pancreatitis advances, persistent stress in the endoplasmic reticulum or oxidative stress hinders autophagic flux, causing autophagosome buildup and the release of DAMPs like reactive oxygen species (ROS) and mitochondrial DNA. These DAMPs activate the NLRP3 inflammasome, triggering the release of proinflammatory cytokines such as IL-1β and intensifying systemic inflammation. Experimental models underscore this duality: β1-synthetase-deficient acinar cells with impaired autophagy induction exhibit heightened AP susceptibility (Ye et al., 2019), while STC-induced autophagic flux blockade exacerbates disease progression (Song et al., 2018). Conversely, rapamycin enhances autophagic flux to attenuate AP in high-fat diet models (Mei et al., 2020), whereas aberrant activation via lncRNA-PVT1/miR-30a-5p/Beclin-1 (Hu et al., 2020) or H2S/AMPK/mTOR (Ji et al., 2016) worsens outcomes. Notably, IL-22 restores autophagy balance via AKT/mTOR activation, mitigating inflammation in caerulein-induced AP (Fu et al., 2024). These findings collectively highlight the intricate and significant relationship between autophagy and AP, underscoring the importance of autophagy regulation in the disease’s progression. Therefore, a comprehensive investigation into the mechanisms regulating autophagy AP, along with the identification of effective targets for autophagy modulation, may offer new perspectives for preventing and treating AP.

The interactions of autophagy-related pathways influencing AP are complex, involving diverse regulators that remain incompletely understood. The mTOR and NF-κB signaling pathways have emerged as focal points of investigation in this domain in recent years. mTOR, part of the PI3K-related kinase family, is a well-preserved serine/threonine kinase. mTOR is a highly conserved serine/threonine kinase that is part of the PI3K-related kinase family. It regulates cell growth, proliferation, energy metabolism, and protein synthesis by integrating extracellular and intracellular signals and activating downstream effector proteins, making it a key regulator of cellular autophagy (Deleyto-Seldas and Efeyan, 2021). AKT, a serine/threonine kinase and a major upstream regulator of mTOR, is recruited to the plasma membrane following PI3K activation. It is essential for mediating autophagy, cell growth, and apoptosis (Zhang et al., 2021b). It was reported that modulation of the AKT/mTOR pathway effectively reduces inflammation and pancreatic tissue damage in SAP model rats, providing a protective effect against SAP (Liu et al., 2025). In addition to the mTOR-related pathway, the role and molecular mechanisms of the NF-κB signaling pathway during autophagy have gained increasing attention. Wan et al. found that the inhibition of the NF-κB signaling pathway during AP led to a reduction in autophagic flux and subsequently mitigated systemic organ damage in mice with AP (Wan et al., 2018). Furthermore, AP is significantly regulated by the IL-9/IL-9 receptor signaling pathway (Gao et al., 2022). In conclusion, targeted modulation of autophagy-related molecular pathways provides a hopeful approach for AP management, providing new opportunities for clinical intervention.

### 3.3 Pyroptosis and AP

Pyroptosis is a caspase-dependent PCD mechanism marked by the creation and rupture of cell membrane pores, cellular swelling, and the release of inflammatory mediators (Gao et al., 2022). Cellular pyroptosis occurs via classical and non-classical pathways. In the classical pathway, diverse external stimuli, such as extracellular ATP, crystalline substances (e.g., monosodium urate or cholesterol crystals), pathogen-associated molecular patterns like bacterial lipopolysaccharide (LPS), and environmental stressors such as ROS, initiate the assembly of NLRP3, pro-caspase-1, and ASC into the NLRP3 inflammasome (Zheng et al., 2023). Activation of the NLRP3 inflammasome activates caspase-1, which cleaves pro-IL-1β into its active form and processes gasdermin D (GSDMD) into its N-terminal fragment (GSDMD-NT). GSDMD-NT inserts into the cell membrane, forming pores that induce cell swelling, rupture, and the release of cellular contents. Inflammatory cytokines IL-1β and IL-18 are also released through these pores, amplifying the inflammatory response and ultimately leading to cell death (Miao et al., 2023). In the non-classical pathway, LPS activates caspase-4/5/11, which cleaves GSDMD to induce cell death, a process distinct from the classical caspase-1-dependent pathway (Xin et al., 2024).

Research has demonstrated a strong association between pyroptosis in pancreatic acinar cells and the progression of AP. During pyroptosis, acinar cells rupture, releasing cellular contents and inflammatory factors, worsening the inflammatory reaction (Al Mamun et al., 2022). GSDMD and NLRP3 are principal mediators of pyroptosis and significantly participate in the pathophysiology of AP (Lv et al., 2022). It has been reported that GSDMD gene deletion in AP model mice significantly inhibited caerulein-induced pyroptosis in pancreatic acinar cells, reduced levels of tumor necrosis factor-α (TNF-α), IL-6, and IL-1β, and attenuated pancreatic necrosis and inflammation. Meanwhile, *in vitro* experiments further confirmed that NLRP3 inhibitors significantly protected acinar cells from pyroptosis injury by inhibiting the GSDMD-dependent pyroptosis pathway (Gao et al., 2021). Furthermore, a separate study revealed that the activation of GSDMD-mediated cellular pyroptosis is critical in the pathogenesis of SAP in a caerulein-induced SAP animal model. Targeted inhibition of GSDMD-mediated pyroptosis reduced the inflammatory response and pancreatic injury in SAP mice (Wu et al., 2021). These results suggest that pyroptosis in pancreatic acinar cells holds significant potential for AP therapy, presenting novel possibilities for the advancement of breakthrough therapeutics for AP.

### 3.4 Ferroptosis and AP

Ferroptosis is a type of cell death that relies on iron and is characterized by the buildup of ROS within cells. It is different in terms of morphology, biochemistry, and genetics from other forms of cell death like apoptosis, pyroptosis, and autophagy (Li J. et al., 2020). Morphologically, ferroptosis is chiefly defined by mitochondrial atrophy, increased mitochondrial membrane density, and degeneration of mitochondrial cristae, without alterations in nuclear morphology or chromatin condensation (Lin et al., 2021). Biochemically, ferroptosis is marked by glutathione (GSH) depletion, reduced glutathione peroxidase 4 (GPX4) activity, ROS accumulation, iron overload, and lipid peroxidation (Bao et al., 2021). Genetically, ferroptosis is controlled by several genes and includes disruptions in iron homeostasis and lipid peroxidation pathways (Xu et al., 2023). Ferroptosis plays a vital role in the development of various diseases, including heart attacks, strokes, and degenerative diseases, and contributes significantly to the progression of AP (Yuan et al., 2021; Li H. et al., 2022).

Studies have shown that two hallmark features of ferroptosis—elevated intracellular iron levels and lipid peroxide accumulation—are intricately associated with oxidative stress during the AP (Li H. et al., 2022). It has been documented that trypsin enhances the susceptibility of pancreatic acinar cells to ferroptosis, aggravating the severity of AP (Liu K. et al., 2022). In a study by Ma et al., lipid ROS and lactate dehydrogenase levels were markedly elevated in cholecystokinin-stimulated acinar cells within an *in vitro* AP model. These changes were reversed by Lip-1, a ferroptosis inhibitor, indicating a pathogenic role for ferroptosis in experimental pancreatitis (Ma et al., 2022). Additionally, ferroptosis is implicated in SAP-related intestinal barrier damage. Inhibition of ferroptosis reduced lipid peroxidation and intestinal barrier damage in SAP, accompanied by increased GPX4 activity and decreased ferroptosis-related gene expression (Ma et al., 2021). Collectively, the above research results show that there is a potential link between ferroptosis and AP.

A substantial body of scientific evidence suggests that targeted modulation of ferroptosis represents an emerging therapeutic strategy for AP. By modulating key targets, this approach can effectively alleviate AP symptoms, promote pancreatic tissue repair, and improve pathological outcomes. GPX4 is an essential protein in the ferroptosis pathway and significantly contributes to lipid peroxidation and cysteine (Cys) metabolism (Liu Y. et al., 2023). GPX4 relies on GSH as a cofactor to reduce phospholipid peroxide production in the cell membrane and convert lipid peroxides into non-toxic lipids. Excessive GSH depletion directly inhibits GPX4 function, disrupting the conversion of toxic lipid peroxides and increasing cellular susceptibility to ferroptosis. However, GPX4 inactivation can directly induce ferroptosis, even when cellular Cys and GSH levels are normal (Zhang et al., 2023). In caerulein-induced AR42J cells, upregulation of GPX4 expression reduced pancreatic acinar cell injury by inhibiting ferroptosis. This was achieved by decreasing intracellular Fe^2+^ levels, restoring the antioxidant system, enhancing GPX and GSH activity, and reducing production of inflammatory factors (Wei et al., 2024). Besides, Ma et al. utilized cerulein to induce AP in mice and demonstrated that targeted modulation of the AP-1/GPX4 pathway substantially protected pancreatic acinar cells from ferroptosis, thereby mitigating pancreatic damage in the mice (Ma et al., 2022). Therefore, targeting and modulating ferroptosis-related signaling pathways may represent an effective therapeutic strategy for AP.

### 3.5 Necroptosis and AP

Necroptosis is a caspase-dependent variant of controlled cellular necrosis that shares characteristics with both apoptosis and necrosis, chiefly marked by cellular swelling and disruption of cell membrane integrity, later triggering inflammatory responses (Yang et al., 2021). This process is initiated by the activation of various death receptors, including tumor necrosis factor receptors (TNFR1, TNFR2), interferon receptors (IFNR), toll-like receptors (TLR3, TLR4), DNA-dependent activators of interferon regulatory factors (DAI), and intracellular RNA or DNA receptors (Pinci et al., 2022). The most well-investigated mechanism among them is the TNF-α/TNFR1 pathway, wherein TNF-α binds to TNFR1 and recruits ubiquitinated receptor-interacting serine/threonine kinase 1 (RIPK1) to form membrane-associated complex I. When caspase-8 activity is reduced or absent, the necroptosis pathway is activated. RIPK1 recruits and phosphorylates RIPK3, which subsequently phosphorylates the pseudokinase mixed-lineage kinase-like (MLKL), translating into a conformational alteration of MLKL. Consequently, necrosomes are formed and translocate to the cell membrane, undermining its integrity, releasing damage-associated molecular patterns, and triggering an inflammatory response (Khoury et al., 2020). Therefore, the RIPK1-RIPK3-MLKL pathway is recognized as the classical necroptosis signaling pathway. Notably, natural metabolites such as emodin, triptolide, dihydromyricetin, and resveratrol have been shown to modulate necroptosis by targeting RIPK1, RIPK3, or MLKL (Zhou et al., 2020a; Zhang et al., 2023a; Sun et al., 2024; Wang et al., 2024b).

Research indicates that necroptosis may represent a critical mechanism underlying acinar cell death in the pathogenesis of AP. An experimental study demonstrated that RIPK3 and MLKL expression is upregulated in the damaged pancreatic tissue of AP mice and caerulein-injured acinar cells. Inhibition of RIPK3 and MLKL expression and phosphorylation significantly reduced pancreatic tissue and acinar cell damage (Yang et al., 2022). In murine AP models, ROS accumulation and elevated expression of RIP3 and phosphorylated MLKL—key necroptosis mediators—were observed in pancreatic tissues and acinar cells. Notably, the inhibitor KN93 suppressed ROS generation and reduced necroptosis-associated protein expression, thereby mitigating acinar cell death (Zhu et al., 2021). Another study reported upregulated RIPK3 and MLKL expression in pancreatic tissues of SAP mice and in acinar cells following rainfrogin intervention, while inhibition of RIPK3/MLKL expression and phosphorylation significantly reduced serum amylase levels and pancreatic histopathological scores in SAP mice (Sun et al. 2022a). Necroptosis also contributes to SAP-associated intestinal and lung injuries, with RIPK3 knockdown alleviating organ damage. Collectively, these studies indicate that RIPK3 and phosphorylated MLKL levels correlate positively with necroptosis severity in AP, and their inhibition mitigates tissue injury (Sundar et al., 2020). In contrast, RIPK3/MLKL-driven necroptosis may exert protective effects in AP pathogenesis. Studies have demonstrated that pancreatic edema and inflammation were exacerbated in RIPK3- and MLKL-deficient mice compared to wild-type controls. Furthermore, MLKL deficiency downregulates anti-apoptotic genes, giving rise to an elevated rate of apoptosis (Boonchan et al., 2021). This finding contrasts with previous research on AP and necroptosis, which reported conflicting outcomes, potentially due to variations in the inducing agent, treatment duration, and timing of necrotic observation (He et al., 2022). However, necroptosis is undeniably linked to the progression of AP. Given these differing roles, further research is required to clarify the relationship between necroptosis and AP.

## 4 Natural products treat AP by regulating PCD

### 4.1 Natural products targeting apoptosis for AP

Numerous studies have demonstrated that certain natural products can alleviate AP and ameliorate pancreatic histopathological injury by regulating pancreatic acinar cell apoptosis and cellular inflammatory responses. Specific pathways involved in the regulation of apoptosis include the NF-κB, MAPK, JAK2/STAT3, and ERS pathways. These findings provide a foundation and insights for applying natural products to treat AP. The underlying mechanisms are summarized in Table 1.

#### 4.1.1 Natural products associated with the NF-κB pathway

Recently, the application of natural products to modulate the NF-κB signaling pathway for disease treatment has garnered significant attention from researchers. Increasing evidence suggests that natural metabolites can inhibit pancreatic acinar cell apoptosis by targeting the NF-κB signaling pathway, highlighting their therapeutic potential for AP. For example, nimbolide, a triterpenoid extracted from the leaves and flowers of *Azadirachta indica* A.Juss. in the family Meliaceae, has been demonstrated in preclinical and mechanistic studies to be non-toxic, non-mutagenic, and to exhibit immunomodulatory, anti-inflammatory, antioxidant, and anticancer properties (Patil et al., 2022). The research conducted by Bansod et al. assessed the impacts of nimbolide on cerulein-induced AP in mice. The results showed that nimbolide pretreatment markedly diminished levels of TNF-α, IL-1β, Bax, and cleaved caspase-3, while upregulating Bcl-2 expression. Furthermore, nimbolide reduced pancreatic acinar cell apoptosis and inflammatory infiltration, with its mechanism of action closely associated with the suppression of the NF-κB pathway (Bansod and Godugu, 2021). However, the absence of a positive control group in the experimental design compromises the reliability of the study’s findings. Crocetin, the principal active metabolite derived from *Crocus sativus* L. of the Iridaceae family, is a carotenoid with a polyunsaturated conjugated enoic acid structure. Research has confirmed that crocetin possesses anti-oxidative stress, hypoglycemic, and anti-apoptotic effects (Liu et al., 2020). Zhu et al. examined the influence of crocetin on caerulein-induced AR42J cells by treating them with varying concentrations of the metabolite. The findings indicated that, in comparison with the control group, crocetin treatment substantially enhanced AR42J cell viability, upregulated Bcl-2 expression, suppressed expression of cleaved caspase-3, CytoC, and Bax, and downregulated the levels of inflammatory factors. Subsequent mechanistic analyses suggested that crocetin’s therapeutic effects on AP may be mediated through the activation of SIRT1 by NF-κB, thereby inhibiting apoptosis and inflammation in pancreatic acinar cells (Zhu and He, 2022). Unfortunately, the study lacked *in vivo* validation to confirm its comprehensive validity, and no positive control group was included in the *in vitro* experimental design, resulting in limited data robustness and reliability. The total flavonoids of Chrysanthemum indicum L. (TFC), which constitute the main active metabolites of the Asteraceae plant *Chrysanthemum indicum* L., are closely related to the traditional therapeutic properties of this plant (Shao et al., 2020). In a study by Yang et al., AP rats were administered TFC via continuous gavage. The results demonstrated that TFC treatment significantly alleviated pancreatic tissue edema, hemorrhage, and necrosis. Additionally, *in vitro* investigations revealed that TFC alleviated cerulein-induced apoptosis in AR42J cells by diminishing the expression of inflammatory factors and apoptotic genes while enhancing anti-apoptotic gene expression via the suppression of the NF-κB signaling pathway (Yang et al., 2024). There were design flaws in the experiment, such as the lack of a positive control group and the failure to identify the specific bioactive metabolites of TFC that caused the therapeutic effects. Liu and colleagues conducted an *in vivo* study to assess daphnetin’s preventive properties in a SAP rat model. Their findings indicated that daphnetin controlled AP inflammation, inhibited pancreatic acinar cell apoptosis, and attenuated pancreatic injury by suppressing the TLR4/NF-κB signaling pathway (Liu Z. et al., 2016). Since there was no positive control group and concentration-dependent response experiments were not conducted, the study’s results are less valid. Tetrandrine is an alkaloid derived from the roots of *Stephania tetrandra* S. Moore, belonging to the Menispermaceae family (Mo et al., 2022). Tetrandrine has been shown to restore pancreatic pathological damage and enhance pancreatic function in STC-induced SAP model rats (Wu et al., 2015). Besides, in an *in vitro* cellular model of LPS-induced AP, pretreatment with anisodamine attenuated pancreatic acinar cell apoptosis and inflammatory responses by inhibiting NF-κB signaling pathways (Li Z. et al., 2020). These findings suggest that natural products may modulate the NF-κB pathway through multiple mechanisms to regulate pancreatic acinar cell apoptosis, offering potential therapeutic strategies for AP.

#### 4.1.2 Natural products associated with the MAPK pathway

The MAPK pathway is a highly conserved signaling cascade in eukaryotic cells, classified within the serine/threonine protein kinase family. Key proteins in this pathway include p38 MAPK, c-Jun N-terminal kinase (JNK), and extracellular signal-regulated kinases (ERK). These proteins are pivotal in regulating apoptosis in AP cells and are integral to AP treatment strategies (Chen X. et al., 2024). Our research indicates that certain natural products can mitigate AP by inhibiting apoptosis in pancreatic acinar cells via the MAPK signaling pathway. Rutaecarpine, a significant alkaloid, is extracted from the Rutaceae plant *Tetradium ruticarpum* (A.Juss.) T. G. Hartley (Li D. et al., 2022). Huang et al. administered rutaecarpine to AP model mice via continuous gavage for 10 days. Compared to the model group, rutaecarpine treatment significantly inhibited the expression of pro-inflammatory cytokines, enhanced anti-inflammatory cytokine levels, and reduced the severity of pancreatic injury in mice. Furthermore, *in vitro* studies demonstrated that rutaecarpine treatment protected against cerulein-induced AR42J cell injury by inhibiting acinar cell apoptosis through the suppression of the MAPK and NF-κB signaling pathways (Huang et al., 2021). However, no toxicity studies or clinical trials were conducted to assess the drug’s safety and therapeutic efficacy. Nobiletin is a polymethoxyflavonoid predominantly present in the peels of citrus fruits, exhibiting a range of bioactive properties, including anti-tumor, antioxidant, anti-inflammatory, anti-obesity, and anti-atherosclerotic effects (Ran et al., 2024). Liu et al. observed elevated levels of TNF-α, IL-1β, and IL-6, along with increased expression of ROS and cleaved caspase-3, in AP mice. Conversely, treatment with nobiletin was found to mitigate oxidative stress, inhibit apoptosis in pancreatic acinar cells, and significantly improve pancreatic histopathology in AP mice, potentially through the inhibition of the p38MAPK pathway (Liu B. et al., 2016). Nonetheless, the study did not include a positive control group or conduct *in vivo* experiments, which limits the robustness and development of the conclusions drawn. Interestingly, different from the studies mentioned above, some natural products have been shown to be able to cause apoptosis in pancreatic acinar cells through the MAPK pathway, suggesting a possible treatment for AP. Ligustrazine, a tetramethylpyrazine alkaloid, is derived from the medicinal plant *Conioselinum anthriscoides* “Chuanxiong” belonging to the Apiaceae family (Li and Gong, 2022). Chen et al. treated caerulein-induced AP rats with ligustrazine, observing that it reduced serum levels of inflammatory factors, upregulated p53 and cleaved caspase-3 expression, and effectively alleviated AP symptoms. *In vitro* studies revealed that ligustrazine reversed caerulein-induced AR42J pancreatic acinar cell injury, probably via the suppression of p38 and ERK MAPK signaling pathways, which promote apoptosis in pancreatic acinar cells (Chen et al., 2016). The study’s conclusions would have been strengthened had the experimental design included a drug concentration-gradient analysis. Escin sodium (ES), a natural combination of triterpene saponins taken from the seeds of *Aesculus hippocastanum* L. of the Sapindaceae family, has also been investigated (Chen R. et al., 2024). *In vivo* and *in vitro* studies demonstrated that ES alleviated STC-induced AP by inhibiting the ERK/STAT3 signaling pathway, which triggers apoptosis in pancreatic acinar cells (Zhang Q. et al., 2021). Therefore, we can find that natural products regulate pancreatic acinar cell apoptosis and mitigate pancreatic inflammation by modulating MAPK-related pathways, offering a novel therapeutic strategy for AP.

#### 4.1.3 Natural products associated with the JAK2/STAT3 pathway

The JAK2/STAT3 signaling pathway is a multifaceted cascade integral to cytokine and growth hormone receptor signaling, significantly influencing the onset and advancement of AP (Zhou Z. et al., 2021). Limonin, a natural tetracyclic triterpenoid, possesses anticancer, anti-inflammatory, and hepatoprotective properties (Fan et al., 2019). Xia et al. investigated the therapeutic effects of limonin in murine models of mild acute pancreatitis (MAP) and SAP. Both models demonstrated elevated levels of IL-6, CCL2, and TNF-α, alongside reduced levels of GSH, SOD, cyclin D1, and Bcl-2. The administration of limonin significantly reversed these alterations, thereby mitigating inflammatory responses and oxidative stress injury in mice with AP. The protective effect is probably enabled by inhibiting the JAK2/STAT3 pathway (Xia et al., 2021). Baicalin is the principal bioactive metabolite isolated from the root of the *Scutellaria baicalensis* Georgi, a member of the Labiatae family (Wang et al., 2022). Through the establishment of an AP mouse model, Yang et al. found that baicalin intervention significantly reduces pancreatic tissue damage and maintains the structural integrity of pancreatic tissue. The underlying mechanism is attributed to the inhibition of inflammatory responses and pancreatic acinar cell apoptosis, which is mediated by the B7H4/JAK2/STAT3 pathway (Yang et al., 2023). However, The study’s limitation is that it used only one drug dose to assess astragaloside’s efficacy in treating acute pancreatitis and lacked a positive control group, preventing a full demonstration of its superiority. Furthermore, the JAK2/STAT3 signaling pathway is implicated not only in pancreatic tissue injury in AP models but also in injuries to other organs caused by AP. Research has illustrated that picroside II treatment reduces inflammatory cytokine levels, mitigates pathological damage in pancreatic and liver tissues, and alleviates SAP-induced hepatocyte apoptosis in rat models by inhibiting the JAK2/STAT3 signaling pathway (Piao et al., 2021). Therefore, the JAK2/STAT3 signaling pathway represents a potential target for natural products in the management of AP and its related issues by inhibiting apoptosis.

#### 4.1.4 Natural products associated with the ERS pathway

When cells are exposed to hypoxia, toxins, oxidative stress, or other environmental perturbations, unfolded or misfolded proteins accumulate in the endoplasmic reticulum, a process termed ERS. Excessive or prolonged ERS can result in cellular dysfunction and apoptosis, ultimately causing orgaregulatory mechanisms. Within the past few years, studies investigating natural products for treating AP by targeting ERS have increased significantly, with growing academic interest in their specific hinging on it as a prominent therapeutic agent for numerous inflammatory conditions. Genistein is a naturally occurring trihydroxyisoflavonoid that has beneficial effects in the treatment of AP (Sharifi-Rad et al., 2021). Xia et al. treated genistein to rats with STC-induced AP in the absence of a positive control group. The results demonstrated that pretreatment with genistein significantly upregulated the expression of glucose-regulated protein 78 (GRP78), caspase-12, protein kinase RNA-like endoplasmic reticulum kinase (PERK), and CHOP proteins; promoted acinar cell apoptosis; and reduced pancreatic inflammatory injury. These effects are likely mediated through the activation of the ERS signaling pathway by genistein, which induces apoptosis (Xia et al., 2019). Quercetin 3-O-xyloside, a flavonoid metabolite derived from sources such as onions, blueberries, apples, and various other fruits and vegetables, is widely recognized for its potent anti-inflammatory and antioxidant properties and establishment of damage (Lee et al., 2016). In a study conducted by Seo et al., rat AR42J cells stimulated with LPS and caerulein were treated with quercetin 3-O-xyloside; however, no positive control group was included in the experimental design. The findings revealed a significant reduction in the levels of ROS, GRP78, and PERK compared to untreated controls. Concurrently, there was a marked increase in the expression of caspase-3 and caspase-9, with these effects being dose-dependent. These results indicate that quercetin 3-O-xyloside confers a protective effect against AP, potentially through mechanisms involving the inhibition of ROS production and the ERS response, thus mitigating apoptosis in pancreatic acinar cells (Seo et al., 2019). Lycopene, a natural fat-soluble carotenoid, exhibits a strong ability to scavenge oxygen free radicals (Khan et al., 2021). The study performed by Lv et al. investigated the impact of lycopene on a SAP model through both *in vivo* and *in vitro* experiments. The findings indicated that lycopene effectively attenuated oxidative stress damage, reduced inflammation, and alleviated SAP symptoms by inhibiting ROS-induced mitochondrial and endoplasmic reticulum stress pathways (Lv et al., 2015). This research has areas for improvement, including the absence of a positive control group and unclear clinical outcomes. Besides, piperine has demonstrated efficacy in the prevention and treatment of AP. In a mouse model of AP induced by L-arginine, piperine was observed to mitigate the pancreatic inflammatory response and reduce pancreatic acinar cell apoptosis. This effect was achieved through the regulation of endoplasmic reticulum stress, which involved the downregulation of IL-1β, caspase-3, and Bax expression, as well as the upregulation of Bcl2 expression (Huang W. et al., 2022). Nonetheless, the experimental validity could have been enhanced by addressing methodological limitations, such as the lack of complementary *in vitro* studies. In conclusion, ERS represents a potential target for natural products in the prevention and management of AP. However, ERS is a complex process involving multiple signaling pathways, and the mechanisms by which natural products target ERS for AP treatment warrant further investigation in the future.

### 4.2 Natural products targeting autophagy for AP

Recent studies have demonstrated that targeted modulation of autophagy may represent a novel therapeutic approach for AP. By influencing autophagy-related pathways and key targets, it can effectively reduce pathological damage, alleviate pancreatic inflammation, and slow the progression of AP. Accumulating evidence suggests that bioactive natural products hold significant potential in targeting autophagy for AP treatment, as summarized below. For an in-depth analysis of the mechanisms, refer to Table 2.

#### 4.2.1 Natural products associated with the NF-κB pathway

The NF-κB signaling pathway plays a critical role not only in apoptosis but also as a key regulator of the cellular autophagic response. Recent studies indicate that specific natural products can alleviate pancreatic inflammation and promote functional recovery by modulating the NF-κB pathway to control autophagy in AP. Salidroside, a bioactive metabolite derived from *Rhodiola rosea* L., a prominent species within the Crassulaceae family, is primarily concentrated in the roots and rhizomes of the plant. Numerous studies have demonstrated that salidroside exhibits a wide range of pharmacological effects, including antioxidant, anti-inflammatory, antifibrotic, and cardioprotective properties (Zhang et al., 2021c). Qian et al. administered salidroside to taurolithocholic acid 3-sulfate-induced AR42J pancreatic acinar cells for 24 h, notably omitting a positive control group. The experimental results revealed that salidroside significantly alleviated pancreatic acinar cell injury and suppressed the release of inflammatory factors. Furthermore, salidroside downregulated the mRNA and protein expression levels of Beclin-1 and LC3-II while upregulating lysosomal-associated membrane protein 2 (LAMP2) expression. This mechanism primarily involves the modulation of the inflammatory response and cellular autophagy processes through the inhibition of the NF-κB signaling pathway (Qian et al., 2021). Another study also demonstrated that salidroside inhibits the NF-κB pathway, enhancing autophagy in pancreatic acinar cells of SAP rats and further delaying SAP progression (Qian et al., 2022). Acanthopanax is obtained from the dried roots, rhizomes, or stems of the plant *Eleutherococcus senticosus* (Rupr. & Maxim.) Maxim, a species belonging to the Araliaceae family. It exhibits various physiological functions, including immune regulation, anti-inflammatory effects, and stress resistance (Zhang X. et al., 2024). Acanthopanax and its positive control, 3-methyladenine, were shown to exert therapeutic effects in a rat model of SAP induced by STC. Mechanistically, Acanthopanax was found to inhibit autophagy in pancreatic acinar cells by modulating the NF-κB signaling pathway, reducing the expression of LC3-II and Beclin-1 at both the protein and mRNA levels, thereby alleviating pancreatic tissue injury (Wang et al., 2016). However, the establishment of a drug concentration-gradient analysis was necessary in this study, which impacted the reliability and validity of the experimental results. Consequently, it is evident that natural metabolites possess the potential to protect pancreatic function and mitigate the symptoms of AP by inhibiting autophagy in pancreatic acinar cells via the regulation of the NF-κB pathway, which might develop into a future option for treating AP.

#### 4.2.2 Natural products associated with the mTOR pathway

The mTOR signaling pathway is an important intracellular mechanism that regulates metabolism, proliferation, and survival, assuming a key role in maintaining cellular homeostasis. Excitingly, mTOR-related targets are essential for the treatment of various diseases, including AP (Gao and Tian, 2023; Ma et al., 2023). Phillygenin, a natural bioactive metabolite classified as a dihydrofuranoid lignan, is primarily found in species of the Oleaceae family, such as *Forsythia suspensa* (Thunb.) Vahl and *Osmanthus fragrans* Lour (Zhou M. et al., 2021). In STC-induced SAP rats, phillygenin pretreatment reduced pancreatic tissue damage, suppressed p62 expression, increased LAMP2 expression, inhibited autophagic vesicle formation, and restored impaired autophagic flux, thus alleviating SAP. Mechanistic studies suggest that this effect may be achieved through inhibition of the PI3K/Akt/mTOR pathway, promoting autophagy in pancreatic acinar cells (Li J. et al., 2024). Since there was no positive control group or concentration gradient comparisons, the effect could not be determined in this investigation. Xanthohumol is an isoprenoid flavonoid metabolite of natural origin, which has demonstrated substantial anti-inflammatory, antioxidant, and immunomodulatory properties (Neumann et al., 2022). Huang et al. found that xanthohumol treatment in SAP mice attenuated pancreatic tissue injury and restored impaired autophagic flux, as evidenced by reduced serum levels of inflammatory factors, decreased malondialdehyde (MDA) expression, increased SOD activity, and decreased expression of p62 and the LC3B-II/LC3B-I ratio. *In vitro* studies further demonstrated that xanthohumol enhances autophagy in pancreatic cells by inhibiting the AKT/mTOR pathway, reducing inflammatory responses and oxidative stress (Huangfu et al., 2023). The aforementioned studies highlight the mTOR pathway as a pivotal component of the autophagic response and a promising target for AP treatment. Consequently, research efforts have concentrated on exploring metabolites that target this pathway for the management of AP.

#### 4.2.3 Natural products associated with other signaling pathway

In addition to the natural products described above, emerging evidence highlights Mogroside IIE as a candidate for AP treatment due to its autophagy-modulating effects via distinct molecular pathways. Mogroside IIE, a naturally sweet triterpenoid saponin primarily derived from the immature fruit of *Siraitia grosvenorii* (Swingle) C. Jeffrey ex A. M. Lu & Zhi Y. Zhang in the Cucurbitaceae family, exhibits multiple pharmacological properties including anti-inflammatory, antioxidant, and metabolic-regulating effects (Cai X. et al., 2021). Xiao et al. employed *in vivo* and *in vitro* experimental AP models to investigate the therapeutic potential of Mogroside IIE. Their results demonstrated that Mogroside IIE mitigates AP progression by reducing p62 and LC3II protein levels, suppressing trypsin and cathepsin B activity. Mechanistically, this effect is attributed to the downregulation of the IL-9/IL-9 receptor-mediated signaling pathway, which regulates aberrant autophagy. However, the absence of a positive control group in this study necessitates further verification of the reliability of the experimental results (Xiao et al., 2020). Collectively, these findings elucidate critical molecular mechanisms for targeting autophagy with natural products in AP treatment. Pathways such as NF-κB, mTOR, and IL-9/IL-9 receptor-mediated signaling may serve as potential therapeutic targets, offering novel directions for future drug development.

### 4.3 Natural products targeting pyroptosis for AP

NLRP3 and GSDMD are key regulators of cellular pyroptosis, making them critical targets for AP treatment and the development of related inhibitors. Several natural products have been discovered as possible therapies for AP by modulating GSDMD- or NLRP3-related pathways. Sinapic acid (SA), a naturally occurring phenolic acid with substantial biological activity, is frequently encountered in fruits, vegetables, and grains (Pandi and Kalappan, 2021). Studies in AP mouse models demonstrate that SA attenuates pyroptosis via inhibition of AMPK phosphorylation and downregulation of NF-κB signaling. This mechanistic action results in reduced expression of GSDMD and NLRP3 inflammasome components, diminished inflammatory cell infiltration, and consequent amelioration of disease severity (Huang Z.-W. et al., 2022). Saikosaponin D (SSD), a bioactive metabolite derived from the Apiaceae plant *Bupleurum chinense* DC., may be used therapeutically for pancreatic disorders (Gu et al., 2025). In a cerulein-induced AR42J cell model, treatment with SSD significantly enhanced cell viability and proliferation while reducing the release of tryptase and pro-inflammatory interleukins. The mechanistic basis for these effects lies in SSD’s ability to mitigate oxidative stress, thereby attenuating mitochondrial damage and inhibiting the cGAS-STING signaling pathway. Collectively, these actions attenuate NLRP3 inflammasome/caspase-1-driven pyroptosis in pancreatic acinar cells, thereby ameliorating cerulein-induced damage in the AR42J model (Chen H. et al., 2024). Unfortunately, the absence of *in vivo* experiments has been identified as a significant methodological limitation, which compromises the translational validity of the findings. Beyond pivotal pathways like GSDMD or NLRP3, certain natural metabolites can alleviate AP by targeting alternative pyroptosis signaling pathways. For instance, salidroside has been demonstrated to significantly reduce pancreatic acinar cell damage and pyroptosis induced by SAP in both *in vivo* and *in vitro* models, effectively inhibiting the release of inflammatory mediators. Western blot and immunohistochemical analyses demonstrated that salidroside’s protective effects are mediated by its ability to inhibit phosphorylation of Akt and NF-κB p65, and to reduce cleaved caspase-3 and GSDME-N-terminal fragment levels. This suggests that salidroside may suppress pyroptosis by inactivating the Akt/NF-κB and Caspase-3/GSDME pathways (Wang X. et al., 2023). However, it is important to acknowledge that the reliability of these findings could have been enhanced by incorporating a concentration gradient in both the *in vivo* and *in vitro* experiments. The specifics of these mechanisms are outlined in Table 2. These findings underscore the therapeutic potential of natural metabolites that modulate pyroptosis signaling pathways, presenting a promising strategy for AP treatment. Further investigation into these mechanisms may lay the groundwork for the development of novel, targeted therapies for this condition.

### 4.4 Natural products targeting ferroptosis for AP

GPX4 is a central and crucial regulatory factor in the mechanism of ferroptosis. Research indicates that deficiency or inhibition of GPX4 in cells leads to lipid peroxidation, inducing ferroptosis, whereas increased levels or activity of GPX4 can inhibit ferroptosis (Xu et al., 2022). Currently, regulating the GPX4 pathway using natural products to inhibit ferroptosis has emerged as a global research focus for treating various diseases. Wedelolactone, a coumarin metabolite primarily derived from *Eclipta prostrata* (L.) L. of the Asteraceae family, exhibits diverse pharmacological activities, including anti-inflammatory, anticancer, neuroprotective, antibacterial, and antiviral effects (Ha et al., 2023). A rat model of STC-induced AP was utilized by Fan et al. to investigate the therapeutic effects of wedelolactone. Wedelolactone treatment was observed to significantly reduce MDA, IL-1β, IL-18, caspase-1, and caspase-11 levels, whereas a marked upregulation was noted in GSH, GPx, and GPX4 expression. The study was further extended through the establishment of an *in vitro* pancreatitis cell model, in which rat AR42J cells were stimulated with LPS and treated with varying concentrations of Wed. The results demonstrated that Wed conferred protection against LPS-induced ferroptosis and pyroptosis in pancreatic acinar cells through modulation of the GPX4 signaling pathway (Fan et al., 2021). Lonicerin, an active metabolite present in *Lonicera japonica* Thunb, exhibits anti-inflammatory and immunoregulatory effects (Lv et al., 2021). Li et al. demonstrated that Lonicerin treatment in an AP cell model reduced Fe^2+^ levels, enhanced Gpx4 expression, and suppressed MDA and ROS production. This effectively inhibited oxidative stress-associated ferroptosis in pancreatic acinar cells and mitigated pancreatic injury, primarily through activation of the SIRT1/GPX4 signaling pathway (Li D. et al., 2024). Nonetheless, the experimental design could be enhanced by incorporating a positive control group, which would more effectively illustrate the treatment’s effectiveness. In addition to AP, natural products may also exert protective effects on AP-associated myocardial injury by modulating ferroptosis. Glycyrrhizin, the primary active metabolite of the Fabaceae plant *Glycyrrhiza glabra* L., was shown by Cui et al. to inhibit the expression of ferroptosis-related genes and to attenuate pancreatic and cardiac tissue injuries in SAP rats. The therapeutic mechanism was mediated through the Keap1/Nrf2/HO-1 pathway, which inhibits ferroptosis (Cui et al., 2024). Table 2 outlines the specific mechanisms through which natural metabolites target ferroptosis for the treatment of AP. These studies suggest that targeting ferroptosis with natural products represents a novel therapeutic strategy for AP, highlighting the need for further exploration.

### 4.5 Natural products targeting necroptosis for AP

At present, there is a paucity of fundamental research on natural products targeting necroptosis of pancreatic acinar cells in the treatment of AP, necessitating further investigation in this area. Fortunately, several metabolites have been identified that attenuate AP by inhibiting macrophage necroptosis. Celastrol, a triterpenoid isolated from the roots of the Celastraceae plant *Tripterygium wilfordii* Hook.f., exhibits anti-inflammatory and antioxidant properties (Wang C. et al., 2023). In a caerulein-induced AP mouse model, celastrol treatment was shown to reduce serum amylase levels, lower histopathological scores, suppress pancreatic p-MLKL expression, and decrease the number of necrotic acinar cells. *In vitro* studies further demonstrated that celastrol attenuated pancreatic macrophage necroptosis through inhibition of the RIPK1/RIPK3/MLK pathway, thereby ameliorating AP (Liang et al., 2023). In addition, baicalin has demonstrated therapeutic efficacy in the treatment of AP (Zhen et al., 2021). While this experiment demonstrated the anti-inflammatory effects of celastrol in the treatment of AP, the absence of a positive control group in both the *in vivo* and *in vitro* experiments limits the ability to fully validate the reliability of the experimental data. Baicalin intervention was reported to significantly reduce cerulein-induced pancreatic acinar cell necrosis and tissue damage scores, as well as reverse pancreatic tissue damage in AP model mice. According to *in vitro* studies, the protective mechanism might be mediated by preventing p-MLKL oligomerization, which would reduce macrophage necroptosis (Huang Y.-T. et al., 2022). However, the toxicological profile and clinical efficacy of baicalin were not addressed in this study, necessitating further investigation to establish its clinical relevance. The above studies have shown that targeting the necroptosis mechanism using natural products represents a promising therapeutic strategy for AP. However, research on natural products targeting pancreatic acinar cell necroptosis remains limited, highlighting the need for further in-depth investigations.

## 5 Discussion

The pathogenesis of AP is intricate, with PCD in pancreatic acinar cells significantly contributing to disease progression. Consequently, pharmacological agents that modulate PCD in pancreatic acinar cells may represent a promising therapeutic strategy for AP management. In recent years, accumulating evidence has demonstrated that natural products exert significant therapeutic effects on AP through targeting pancreatic acinar cell PCD. Based on these findings, this review systematically compiles and analyzes recent research advances in natural products targeting PCD in pancreatic acinar cells for AP treatment, thereby providing novel insights for future prevention and therapeutic interventions of AP.

The different forms of PCD are interrelated rather than entirely isolated. Caspase-3, a multifunctional protease, is essential in apoptosis and also cleaves the pyroptosis-related GSDME, consequently enhancing apoptosis (Kist and Vucic, 2021). Similarly, caspase-8 extends its function beyond apoptosis, significantly contributing to both necroptosis and pyroptosis pathways (Zhang W. et al., 2024). GPX4 serves as a critical regulator of ferroptosis, and its deficiency is associated with the initiation of cellular pyroptosis (Wang L. et al., 2023). The induction of ferroptosis is connected to LC3 conversion and the combination of autophagosomes with lysosomes. Conversely, the elimination of essential proteins and genes within the autophagy pathway can inhibit cell death by activating the ferroptosis pathway (Tang et al., 2019). In AP, the simultaneous occurrence of multiple forms of PCD during disease progression has enhanced our understanding of its underlying mechanisms and therapeutic strategies. In an *in vitro* model of LPS-induced SAP, miR-20b-5p was found to attenuate inflammatory progression by suppressing apoptosis through autophagy enhancement, suggesting an inhibitory role of autophagy in apoptotic signaling (Tang et al., 2021). *In vivo* studies further revealed that propylene glycol alginate sodium sulfate alleviates pancreatic injury in cerulein-induced AP mice via activation of the MEK/ERK pathway. This intervention upregulated the anti-apoptotic protein Bcl-2 and suppressed Beclin-1-mediated autophagy through strengthened Bcl-2/Beclin-1 interactions, implicating this molecular crosstalk as a key regulatory node between apoptosis and autophagy (Zhang et al., 2017). Notably, concurrent inhibition of apoptosis and ferroptosis by Hspb1 has been shown to mitigate acinar cell damage and prevent severe AP progression, underscoring the therapeutic potential of dual-targeting PCD pathways (He et al., 2024). In addition, studies have shown that ferroptosis-related genes are regulated by apoptosis and necroptosis, with multiple cell death modes coexisting and interacting to influence AP progression (Li J. et al., 2023). These findings underscore that AP progression involves dynamic, context-dependent crosstalk among PCD pathways. To elucidate the interactive networks of PCD pathways, integrated systems biology approaches—combining multi-omics and network pharmacology—demonstrate unique advantages. In a study on cinnamaldehyde (CA) for gastric mucosal injury, transcriptomic analysis showed CA affects apoptosis, autophagy, ferroptosis, and the PI3K-AKT pathway by altering 1,302 genes. KEGG analysis identified PI3K-AKT as a key hub for pathway interactions (Yan et al., 2024). In curcumin’s anti-HCC research, network pharmacology highlighted 167 key pathways, with experimental validation showing activation of apoptosis and autophagy (Chen et al., 2022). Transcriptomics provides genome-wide regulatory insights, while network pharmacology pinpoints critical pathway hubs, together establishing a robust framework for natural product-driven multi-PCD targeting.

Beyond canonical PCD pathways, recent discoveries of non-canonical cell death modalities are reshaping our understanding of AP pathophysiology. Parthanatos, a caspase-independent cell death mechanism driven by poly (ADP-ribose) polymerase-1 (PARP-1) overactivation and mitochondrial dysfunction, may contribute to AP due to PARP-1’s role in increasing oxidative stress (Nagy-Pénzes et al., 2022). However, direct evidence linking PARP-1 to pancreatic acinar cell death is still lacking. Additionally, cuproptosis, a copper-dependent cell death process triggered by mitochondrial stress, is proposed as a potential factor, with studies showing that copper chelators can reduce acinar cell death in murine models of L-arginine-induced AP (Xue et al., 2023). Nonetheless, the hypothesis that copper overload worsens pancreatic injury through cuproptosis has not been experimentally validated. Parthanatos and cuproptosis, less explored forms of PCD, highlight opportunities for new therapies but require validation. Advancing AP treatment needs a deeper understanding of known PCD mechanisms and systematic study of new pathways to develop precise, multi-targeted interventions.

Natural product-targeted PCD has shown significant therapeutic benefits in both preventing and treating AP. The key advantage of these natural products lies in their ability to synergistically regulate multiple targets. For instance, lonicerin specifically activates the SIRT1/GPX4 signaling pathway, enabling dual modulation of apoptosis and ferroptosis. In contrast, crocetin inhibits the NF-κB signaling pathway, inducing synergistic anti-inflammatory and anti-apoptotic effects. This multi-pathway intervention strategy demonstrates therapeutic superiority over single-target synthetic drugs. Moreover, certain natural products exhibit distinct tissue selectivity. For example, ligustrazine induces apoptosis in pancreatic cells in AP models while conferring anti-apoptotic protection to liver and kidney cells (Li et al., 2017). This dual functionality addresses the systemic limitations of traditional drugs. Recent advances in exploring the biological diversity of natural product sources have expanded opportunities for therapeutic innovation. Beyond plant-derived metabolites, animal-sourced flavonoids, such as propolis-derived pinocembrin, effectively mitigate AP by suppressing oxidative stress, inflammatory responses, and apoptosis (Ali et al., 2024). Similarly, the gut microbiota metabolite urolithin A impedes SAP progression by modulating endoplasmic reticulum-mitochondrial calcium channels and reducing necroptosis (Kang et al., 2024). These properties underscore the unique therapeutic potential of natural products in terms of target specificity, safety, and pathological adaptability.

However, the development, utilization, and clinical translation of natural products face substantial challenges. Firstly, many natural products have complex compositions, extraction difficulties, and multifaceted mechanisms of action, resulting in challenges for standardization and quality control. Future research should prioritize identifying bioactive agents through systematic screening and developing efficient, selective, and eco-friendly extraction technologies. Furthermore, challenges such as poor solubility, low bioavailability, unpredictable metabolic conversion, and safety concerns substantially hinder druggability. Structural optimization strategies could improve bioavailability, enhance targeting, and minimize toxicity. Studies have demonstrated that the resveratrol derivative (R)-TML104 exhibits enhanced bioavailability and metabolic stability in controlling AP by suppressing interleukin-6-driven inflammation through AMPK/SIRT1 activation, providing a novel therapeutic strategy for pancreatic injury (Ren et al., 2022). Besides, a dithioketal-modified liposomal nanoparticle system engineered for the delivery of kaempferol (KA) has been developed to optimize mitochondrial homeostasis within the framework of SAP therapy. This system activates Nrf2 to enhance redox equilibrium and modulates mitochondrial dynamics through dynamin-related protein 1 and PTEN-induced kinase 1 pathways. Consequently, this approach significantly augments the antioxidant efficacy, bioavailability, and safety profile of KA (Wen et al., 2024). Despite these advancements, clinical evidence remains limited, especially regarding their regulation of PCD. For instance, while Euphorbia kansui (Mo et al., 2019) and nano-curcumin (Chegini et al., 2023) reduced inflammation or hospital stays in AP patients, neither study examined PCD mechanisms. Similarly, a pentoxifylline trial (Vege et al., 2020) showed no clinical benefit and ignored PCD biomarkers. These findings collectively reveal two key issues: 1) an overemphasis on general anti-inflammatory effects rather than PCD-specific pathways, and 2) methodological limitations (small cohorts, single-center designs) reducing reliability. Thus, while natural products exhibit multi-modal therapeutic potential, their PCD-specific roles in AP remain clinically unproven.

## 6 Conclusion and future perspectives

This review highlights the mechanisms of PCD in AP and identifies natural products as potential therapies targeting PCD in pancreatic acinar cells, yet three key limitations remain. First, the complex composition and lack of standardization of natural products complicates mechanistic studies, as research predominantly examines single metabolites or extracts targeting specific pathways, neglecting systematic analysis of multitarget synergies. Second, basic research disproportionately focuses on apoptosis and autophagy, while ferroptosis, pyroptosis, and necroptosis pathways are underexplored. Third, clinical translation is hindered by inadequate evaluation of pharmacokinetic challenges (e.g., bioavailability limitations, metabolic instability) and potential toxicity in standardized trials, limiting practical applicability.

To address these challenges, future research should prioritize: 1) Elucidate PCD interaction networks using multi-omics approaches (e.g., transcriptomics, proteomics) to map the dynamic interplay between caspase-3/8, Bcl-2/Beclin-1, and other molecular regulators; 2) Develop multi-target strategies integrating computational pharmacology tools (e.g., molecular docking, AI-driven drug design) to engineer natural product derivatives targeting critical nodes like GPX4 and GSDME; 3) Optimize delivery systems using nanocarriers (e.g., liposomes, exosomes) to improve tissue-specific targeting, metabolic stability, and pharmacokinetic profiles; and 4) Advance clinical translation by curating standardized natural product databases with validated quality control metrics and conducting multicenter randomized controlled trials to evaluate long-term efficacy and safety.

In summary, while natural products targeting PCD offer promising therapeutic avenues for AP, clinical translation remains nascent. Realizing their potential requires interdisciplinary efforts to address standardization hurdles, advance mechanistic understanding, optimize technical precision, and accelerate translational research—essential steps for developing effective AP therapies.
